# Parent of origin gene expression in the bumblebee, *Bombus terrestris*, supports Haig's kinship theory for the evolution of genomic imprinting

**DOI:** 10.1002/evl3.197

**Published:** 2020-10-12

**Authors:** Hollie Marshall, Jelle S. van Zweden, Anneleen Van Geystelen, Kristof Benaets, Felix Wäckers, Eamonn B. Mallon, Tom Wenseleers

**Affiliations:** ^1^ Department of Genetics and Genome Biology The University of Leicester, Leicester LE1 7RH United Kingdom; ^2^ Laboratory of Socioecology and Social Evolution Department of Biology, KU Leuven, 3000 Leuven Belgium; ^3^ Biobest Belgium N.V., 2260 Westerlo Belgium; ^4^ The Lancaster Environmental Centre University of Lancaster, Lancaster LA1 4YW United Kingdom

**Keywords:** Bumblebee, genomic imprinting, Hymenoptera, kinship theory, reciprocal cross, RNA‐seq

## Abstract

Genomic imprinting is the differential expression alleles in diploid individuals, with the expression being dependent on the sex of the parent from which it was inherited. Haig's kinship theory hypothesizes that genomic imprinting is due to an evolutionary conflict of interest between alleles from the mother and father. In social insects, it has been suggested that genomic imprinting should be widespread. One recent study identified parent‐of‐origin expression in honey bees and found evidence supporting the kinship theory. However, little is known about genomic imprinting in insects and multiple theoretical predictions must be tested to avoid single‐study confirmation bias. We, therefore, tested for parent‐of‐origin expression in a primitively eusocial bee. We found equal numbers of maternally and paternally biased expressed genes. The most highly biased genes were maternally expressed, offering support for the kinship theory. We also found low conservation of potentially imprinted genes with the honey bee, suggesting rapid evolution of genomic imprinting in Hymenoptera.

**Impact Summary**

Genomic imprinting is the differential expression of alleles in diploid individuals, with the expression being dependent on the sex of the parent from which it was inherited. Genomic imprinting can be viewed as an evolutionary paradox. Natural selection, in most cases, is expected to favor expression of both alleles to protect against recessive mutations that render a gene ineffective. What then is the benefit of silencing one copy of a gene, making the organism functionally haploid at that locus? Several explanations for the evolution of genomic imprinting have been proposed. Haig's kinship theory is the most developed and best supported.

Haig's theory is based on the fact that maternally and paternally inherited alleles in the same organism can have different interests. For example, in a species with multiple paternity, a paternal allele has a lower probability of being present in siblings that are progeny of the same mother than does a maternal allele. As a result, a paternal allele will be selected to value the survival of the organism it is in more highly compared to the survival of siblings. This is not the case for a maternal allele.

Kinship theory is central to our evolutionary understanding of imprinting effects in human health and plant breeding. Despite this, it still lacks a robust, independent test. Colonies of social bees consist of diploid females (queens and workers) and haploid males created from unfertilized eggs. This along with their social structures allows for novel predictions of Haig's theory.

In this article, we find parent‐of‐origin allele‐specific expression in the important pollinator, the buff‐tailed bumblebee. We also find, as predicted by Haig's theory, genes showing matrigenic and patrigenic bias involved in reproduction with the most extreme bias been found in matrigenically biased genes.

Genomic imprinting is the differential expression of alleles in diploid individuals, with the expression being dependent on the sex of the parent from which it was inherited (Pegoraro et al. 2017). Multiple evolutionary theories attempt to explain its existence (reviewed in Patten et al. 2014). The most widely accepted explanation is the kinship theory developed by Haig Haig (2000). This theory predicts genomic imprinting arose due to natural selection acting differently on the maternal alleles and the paternal alleles of an individual for given processes. For example, in a polyandrous mating system with maternal care (e.g., mammals), paternal alleles are predicted to be subject to selection pressures that increase resource allocation from the mother at the expense of siblings, whereas maternal alleles in this scenario are predicted to be selected for more equal resource distribution amongst offspring.

The majority of support for this theory comes from studies based on mammals and flowering plant systems (Patten et al. 2014). However, it has been suggested haplodiploid social insects can provide an ideal system to independently test Haig's kinship theory (Queller 2003). Colonies of social bees consist of diploid females (queens and workers) and haploid males created from unfertilized eggs. This along with their social structures allows for novel predictions of Haig's theory.

Research exploring parent‐of‐origin effects in social insects has focused on the behavioral and physiological outputs of genetic crosses. In the Argentine ant (*Linepithema humile*) paternal effects were observed in care‐giving associated behaviors and in sex allocation of offspring (Libbrecht et al. 2011; Libbrecht and Keller 2012). Paternal effects on dominance and stinging behavior have also been observed in crosses of European and Africanized honey bees (Guzman‐Novoa et al. 2005). Additionally, Oldroyd et al. (2014) found a parent‐of‐origin effect of increased ovary size in honey bees but could not definitively determine which parent this effect was driven by.

More recently, reciprocal crosses and next‐generation sequencing technologies have been used to identify genes with parent‐of‐origin allele‐specific expression patterns in honey bees (Kocher et al. 2015; Galbraith et al. 2016). Both groups used RNA‐Seq to study parent‐of‐origin gene expression in hybrid crosses of honey bee subspecies. The logic of their test was that as honey bee queens are multiply mated, maternal alleles can occur in half sisters and therefore should be selected to moderate worker reproduction in queenless colonies. Paternal alleles, on the other hand will not be in half sisters and will be selected to reproduce at any cost (Galbraith et al. 2016). Therefore, the prediction is that parent‐of‐origin allele‐specific expression should exist in honey bees and patrigenic expression will dominate in reproductive workers (Galbraith et al. 2016). Surprisingly, Kocher et al. (2015) found a matrigenic bias in gene expression however, it was later shown that subspecies incompatibility effects influenced the results obtained (Gibson et al. 2015). Showing support for the kinship theory, Galbraith et al. (2016) found greater patrigenic expression in reproductive workers compared to sterile workers, with increased patrigenic expression in the reproductive tissues.

The lack of agreement between these studies weakens their support for Haig's theory. Another weakness of the evidence is that it is limited to only one species and tests only one prediction from the many predictions made Queller (2003) for Haig's theory and genomic imprinting's role in social insect biology.

To test the robustness of Haig's kinship theory we present gene expression data (RNA‐seq) of reproductive and sterile workers from reciprocal crosses of subspecies of the primitively eusocial bumblebee, *Bombus terrestris*. As in Galbraith et al. (2016) we also test the effect of queenless conditions, however this species is naturally singly mated. As such, worker daughters share their entire paternal genomes and are therefore equally related to sons and nephews (Fig. 1). Under queenless conditions for singly mated social insects the kinship theory predicts that paternal alleles will be selected to equally favor reproduction and sterility as the likelihood of the paternal alleles being passed on in a son or nephew is equal. However, individual workers are more closely related to their sons compared to their nephews regarding maternal alleles (Fig. 1). Under the same conditions the kinship theory now predicts maternal alleles will be selected to favor worker reproduction as there is a higher likelihood of a given maternal allele being inherited by a son than by a nephew. It is therefore expected that genes involved in reproductive processes will be imprinted and under the scenario detailed above it will be the maternal copy of the reproductive genes which will be more highly expressed. These predictions for the presence of matrigenic/patrigenic expression bias are different from those predicted for the naturally multiply mated honey bee (Queller 2003). Under a multiple mating scenario, it is the paternal alleles that should favor worker reproduction. Therefore we make four predictions: (1) parent‐of‐origin allele‐specific expression exists in bumblebees, (2) genes showing both maternal and paternal allele‐specific expression bias will be present, (3) genes showing parent‐of‐origin expression will be enriched for reproductive‐related processes, and (4) maternal expression bias will be higher in reproductive workers for genes involved in reproduction.

**Figure 1:**
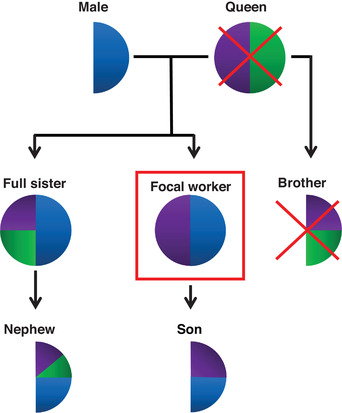
Inheritance probabilities for a given gene under queenless conditions in bumblebees. As the bumblebee is singly mated and males are haploid, there is only one paternal allele (blue) shared between all female workers. This means there is an equal probability of the paternal allele ending up in the worker's son or nephew. In this example, the focal worker has inherited the purple maternal allele. Her sister has a 50% change of inheriting the purple or the green maternal allele. For the purple maternal allele, there is a 50% chance of ending up in the son but a 25% chance of ending up in the nephew. The kinship theory therefore predicts maternal alleles (i.e., the purple gene in the focal worker) to be selected to favor reproduction at the cost of sisters as there is a lower probability that the given maternal allele will be present in the offspring of sisters, whereas paternal alleles (blue) should not be under strong selection to either increase reproduction or maintain sterile 'helping' behavior. Schematic adapted from Drewell et al. (2012).

## Methods

### SAMPLE COLLECTION

**Figure 2:**
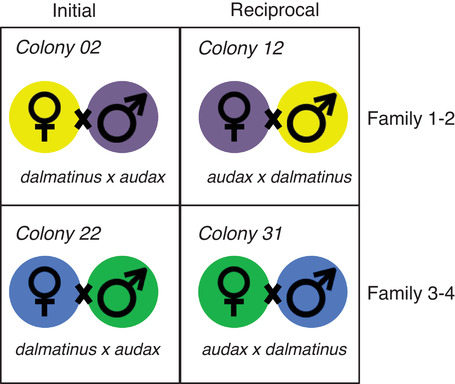
Graphic display of the family‐wise reciprocal crosses carried out. Each color refers to related individuals, that is, the queen from colony 02 is the sister of the male used in colony 12. This design reduces genetic variability between the initial and reciprocal crosses allowing parent‐of‐origin expression to be disentangled from allele‐specific expression caused by the genotype.

Reciprocal crosses of *B. terrestris dalmatinus* (native to southern Europe) and *B. terrestris audax* (native to the United Kingdom) were carried out by Biobest, Leuven. Reciprocal crosses allow subspecies‐of‐origin effects (i.e., the effect of genotype) to be disentangled from parent‐of‐origin effects (Kocher et al. 2015; Galbraith et al. 2016). To obtain enough successful colonies multiple males and females for each cross (Fig. 2) were released into cages to mate. Once mating had occurred, the males and females were removed. The males were immediately frozen at −80${}^{\circ }$C and the females were placed in cold conditions for eight weeks to induce diapause. Ten matings were carried out for each cross.

Four successful colonies (one of each cross‐direction) from two “families” (Fig. 2), resulting in two replicate crosses, were housed at the University of Leuven and kept in 21${}^{\circ }$C with red‐light conditions, they were fed ad libitum with pollen and a sugar syrup. Isogenic lines of bumblebees do not exist as they suffer from inbreeding issues, however we used siblings for the crosses (shown as families in Fig. 2) to maximize homozygosity between the male of one cross and the female of the reciprocal cross. Crossing two different homozygous lines (subspecies in this case) increases the likelihood of F1 progeny being heterozygous at a given locus, allowing us to determine the parental origin of each allele. By creating reciprocal crosses we can disentangle the effect of genotype on allele‐specific expression. If genotype drives allele‐specific expression this would manifest as lineage‐of‐origin expression in our model, that is, high expression of the allele in the male of one cross and the female of the reciprocal cross, as they share the same genetic background. Parent‐of‐origin expression can be identified when high expression of an allele is always attributed to the maternal/paternal allele irrespective of differences in the underlying genotype.

Callow workers (individuals <24‐hour‐old) were labeled with numbered disks to determine age and allow behavior to be recorded. Once each colony contained approximately 30 workers, the queen was removed. The colonies were then filmed under queenless conditions for 30 minutes per day for 14 days to score individual behavior. The following behaviors were used to classify workers: incubating, feeding larvae, inspecting brood cells, building egg cups, ventilation, biting, pushing, egg‐laying, egg‐eating, foraging, feeding, and grooming. Workers were classified based on the frequency of each of the above behaviors as either sterile foragers, sterile nurses, dominant reproductives or subordinate reproductives (Supporting Information 1.0.0).

Worker reproductive status was confirmed by ovary dissection, ovaries were scored on a 0–4 scale as in Duchateau and Velthuis Duchateau and Velthuis (1988), entire bodies were then stored at −80${}^{\circ }$C along with the original queen mothers and male fathers. Workers were selected for sequencing based on their behavioral classification and ovary status. Two of each behavioral type per colony were selected (i.e., two dominant reproductives, two subordinate reproductives, two nurses, and two foragers), with the exception of colony 22 (Fig. 2) that contained three subordinate reproductives and one dominant reproductive. We chose to sample in this way to ensure we capture the range of behavioral differences shown by *B. terrestris* within our data. All sterile and reproductive samples were age‐matched. This gave a total of 32 samples, 8 per colony, 4 of each reproductive status, reproductive or sterile; see Supporting Information 1.0.0 for behavioral and ovary scoring per sample.

### DNA AND RNA EXTRACTION AND SEQUENCING

DNA was extracted from the mother and father of each colony using the Qiagen DNeasy® Blood & Tissue Kit. Thirty‐two workers were selected for RNA sequencing as described above. The head and abdomen were dissected and RNA extracted separately for each, using the Qiagen RNeasy® Lipid Tissue Kit, giving 64 total RNA samples. The quality of the DNA and RNA extraction were measured by Nanodrop and Qubit® fluorometer. Whole genome parental DNA was sequenced using 91 bp paired‐end reads with an insert size of 500 bp, and worker RNA was sequenced using 90 bp paired‐end reads with an insert size of 200 bp, on an Illumina HiSeq 2000 by BGI, China, in 2014 using BGI's standard library preparation protocols. Lane effects were minimized for the RNA samples by spreading colony, tissue, and worker‐type samples across five lanes to ensure that a single variable was not sequenced on a single lane adding a confounding factor.

### GENERATION OF ALTERNATIVE REFERENCE GENOMES

BGI provided whole genome sequencing data with trimmed low‐quality reads and adapters. We quality checked the data using Fastqc version 0.11.05 (Andrews 2010) and trimmed 10 bp from the start of each read, using Cutadapt version 1.11 (Martin 2011) as base composition bias was present, this was likely introduced by the previous aggressive adapter removal. Reads were aligned to the bumblebee reference genome (Bter_1.0, Refseq accession no. GCF_000214255.1; Sadd et al. 2015) using BWA‐mem version 0.7.15 (Li and Durbin 2009) with standard parameters. The mean alignment rate of reads was 98.0% ± 0.3% (mean ± SD), this resulted in a final coverage of 14.3*X* ± 1.0*X* (Supporting Information 1.0.1). Single‐nucleotide polymorphisms (SNPs) were then called using freebayes version 1.1.0 (Garrison and Marth 2012), which can account for the difference in ploidy between males and females, on individual samples with a minimum count of two observations for alternate alleles and a minimum coverage of five reads per SNP. SNPs were then filtered to keep only those with a minimum quality score of 20. Queen SNPs were also filtered so only the homozygous alternative SNPs remained. The subtracted command from BEDtools version 2.25.0 (Quinlan and Hall 2010) was then used to create files containing SNPs unique to either the mother/father of each colony, as in Galbraith et al. (2016). The individual parental SNP files were then used to create alternate reference genomes for each parent using the “fasta alternate reference maker” command in GATK version 3.6 (McKenna et al. 2010). This method allows us to confidently allocate reads to either the maternal or paternal chromosome meaning we can assess the relative expression level of each parental allele.

### IDENTIFICATION OF PARENT‐OF‐ORIGIN EXPRESSION

BGI provided RNA‐Seq data with trimmed low‐quality reads and adapters. We quality checked and trimmed the data as above. STAR version 2.5.2b (Dobin et al. 2016) was used to align worker RNA‐seq reads to each of that colony's specific parental genomes with zero mismatches allowed. This ensures any reads containing a SNP will only be matched to the parent that allele was inherited from. Alignment files were then filtered using the intersect feature from BEDtools version 2.25.0 (Quinlan and Hall 2010), so only alignments that contain an informative SNP (a unique SNP from either the mother or father) were kept, this method allows us to allocate reads to either the maternal or paternal allele. Reads were counted for the maternal/paternal alignments, also using BEDtools version 2.25.0 (Quinlan and Hall 2010) and SNP positions were annotated with a gene ID taken from the Bter_1.0 annotation file (Refseq accession no. GCF_000214255.1) using a custom R script. SNPs that had zero maternal reads in at least one sample were removed completely from the analysis to avoid possible inflation of paternal counts. This would occur if the queen position was imscalled as homozygous with the missing allele matching that of the male (Galbraith et al. 2016).

Genes showing parent‐of‐origin expression were determined using a logistic regression model in R version 3.4.0 (https://cran.r-project.org). Only genes occurring in both cross‐directions and in both family combinations, with a minimum of two SNPs per gene were analyzed, this left a total of 7508 genes. If any gene showed zero reads for paternal counts, this was changed to 1 to avoid complete separation. A quasibionimal distribution was also used to account for overdispersion within the data. Fixed factors included the direction of the cross, family, and reproductive status (reproductive or sterile). Correction for multiple testing was carried out using the Benjamini‐Hochberg method (Benjamini and Hochberg 1995). Genes were determined as showing parent‐of‐origin expression if the allelic ratio (maternal/paternal) corrected p‐value was <0.05 and the parental expression proportion was >0.6.

### DIFFERENTIAL EXPRESSION

All RNA‐seq samples were aligned to the reference genome (Bter_1.0, Refseq accession no. GCF_000214255.1; Sadd et al. 2015) using STAR version 2.5.2b (Dobin et al. 2016) with standard parameters. HTseq version 0.8.0 (Anders et al. 2013) was then used to count the number of reads per gene for each sample. Differential gene expression between reproductive and sterile workers for head and abdomen samples was assessed using the DESeq2 package version 1.16.1 (Love et al. 2014) in R. Data were filtered to remove genes with low expression, <10 counts and counts were rlog transformed to reduce differences between samples with low counts and to normalize by library size. DESeq2 allows the incorporation of a general linear model, which incorporates estimates of size factors and data dispersion, to identify differential expression; family, age, weight, direction of the cross, tissue type, and reproductive status were all factors. *P*‐values were corrected for multiple testing using the Benjamini‐Hochberg method (Benjamini and Hochberg 1995).

### GENE ONTOLOGY ENRICHMENT

GO enrichment analysis was carried out using the hypergeometric test with Benjamini‐Hochberg (Benjamini and Hochberg 1995) multiple‐testing correction (q<0.05) in a custom R script implementing the R package GOstats (Falcon and Gentleman 2007). This script used GO annotations previously created in Bebane et al. (2019) for the *B. terrestris* genome, Bter_1.0. GO terms for differentially expressed genes were tested for enrichment against GO terms associated with all genes identified in either the RNA‐seq data from the abdomen or head. Upregulated genes in either reproductive or sterile workers were tested for GO term enrichment against all differentially expressed genes from the respective tissue type as a background set.

Genes showing parent‐of‐origin expression were tested for enrichment against GO terms associated with all genes identified in both abdomen and head RNA‐Seq data sets. Genes maternally or paternally biased were checked for GO term enrichment against all genes showing parent‐of‐origin expression as a background set. REVIGO (Supek et al. 2011) was used to obtain the GO descriptions from the GO identification numbers.

### COMPARATIVE ANALYSES

A hypergeometric test was applied to gene lists from the differential expression analysis and the parent‐of‐origin expression analysis to identify potential enrichment. *Bombus terrestis* and *Apis mellifera* orthologous genes were determined in Marshall et al. (2019), briefly, a reciprocal blast was carried out between the honey bee (Amel_4.5, Refseq accession no. GCA_000002195.1) and bumeblebee (Bter_1.0, Refseq accession no. GCA_000214255.1) genomes, with a minimum *e*‐value of $1\times \;10^{-3}$ and allowing only one match per gene. Only genes that matched in both directions and to the same gene were kept. A custom R script was then used to check for overlap between genes identified as showing parent‐of‐origin expression here and orthologous *A. mellifera* genes identified in Galbraith et al. (2016).

## Results

### PARENT‐OF‐ORIGIN GENE EXPRESSION

We mapped all RNA‐Seq data to the maternal and paternal parental genomes with zero mismatches. Even though alignment rates decreased, we still maintained a large number of reads for subsequent analysis. The mean number of uniquely mapped reads to the maternal genomes was 91.4% ± 1.3% (mean ± SD), averaged across the 64 RNA‐Seq libraries. This equated to a mean of 13,919,441 ± 2,172,427 uniquely mapped reads (Supporting Information 1.0.1). The mean number of uniquely mapped reads to the paternal genomes was 91.4% ± 1.4% (mean ± SD), averaged across the 64 RNA‐Seq libraries. This equated to a mean of 13,891,964 ± 2,169,676 uniquely mapped reads (Supporting Information 1.0.1).

A total of 10,211 genes had a minimum of two SNPs with at least a coverage of five reads each, the median number of SNPs per gene was 13. Of those, 7508 genes occurred in every cross, worker type, and tissue type. Seven hundred genes in reproductive workers significantly deviated from the expected 0.5 allelic expression proportion in the same direction (i.e., higher maternal or paternal expression), in both initial and reciprocal cross, in both replicate crosses, and so show significant maternal/paternal expression bias (q<0.05). Seven hundred forty‐seven genes show the same significant deviation from 0.5 for sterile workers. The expression bias was averaged across: tissue type, worker type, family, and direction of cross to obtain an extremely conservative expression proportion, resulting from a total of 32 RNA‐Seq libraries per gene per reproductive state (two tissues from four individuals per reproductive state from four colonies total). The significant genes were then filtered to also have an average maternal expression proportion of >0.6 or <0.4 to give a final confident list of genes showing parent‐of‐origin expression (Supporting Information 2, Fig. S1). We also found the significant genes with the highest proportion of maternal or paternal expression bias also showed low variation between tissues and colonies (Supporting Information 2, Fig. S2).

**Figure 3:**
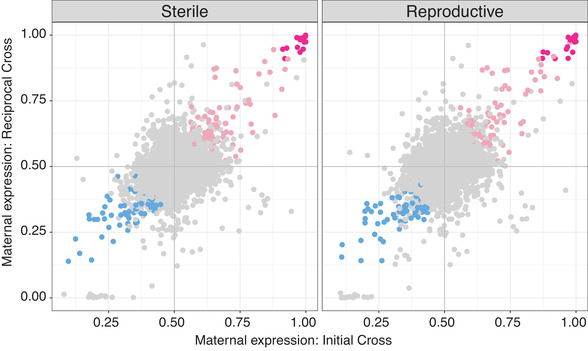
Maternal expression proportion of all genes by worker reproductive state. Each point represents a gene. Blue points are genes with significant paternal expression bias (q<0.05 and maternal expression proportion <0.4). Pink points are genes with significant maternal expression bias (q<0.05 and maternal expression proportion >0.6). Dark pink points are genes with significant maternal expression bias (q<0.05) with the proportion of maternal expression >0.9. The top‐left quadrant of each plot represents genes with a *B. terrestris audax* expression bias, the bottom‐right quadrant represents gene with a *B. terrestris dalmatinus* expression bias. The top‐right represents genes with a maternal expression bias and the bottom‐left represents genes with a paternal expression bias.

Reproductive workers have 163 genes showing significant parent‐of‐origin expression (q<0.05, expression proportion >0.6, Fig. 3) (Supporting Information 1.1.0). Sterile workers have 170 genes showing significant parent‐of‐origin expression (q<0.05, expression proportion >0.6, Fig. 3) (Supporting Information 1.1.1). There is no significant difference between the number of genes showing maternal expression bias compared to paternal expression bias based on reproductive status, that is, reproductive and sterile workers show similar numbers of maternal and paternal biased genes (chi‐squared test of independence, $\chi^{2}$ = 0, df = 1, p‐value = 1). There is also no difference in the number of genes showing paternal expression bias compared to maternal expression bias in reproductive and sterile workers, assessed independently, that is, the equal number of genes was found to show maternal and paternal expression bias in both reproductive phenotypes (chi‐squared goodness of fit, reproductive: $\chi^{2}$ = 1.3804, df = 1, p‐value = 0.24, sterile: $\chi^{2}$ = 1.5059, df = 1, p‐value = 0.2198). The most extreme expression bias is seen in the maternally expressed genes in both phenotypes, with 17 genes showing a maternal expression proportion of >0.9 in both reproductive and sterile workers (Fig. 3). There were no genes showing >0.9 paternal expression bias. Additionally, we did not find any genes with significant subspecies expression bias.

**Figure 4:**
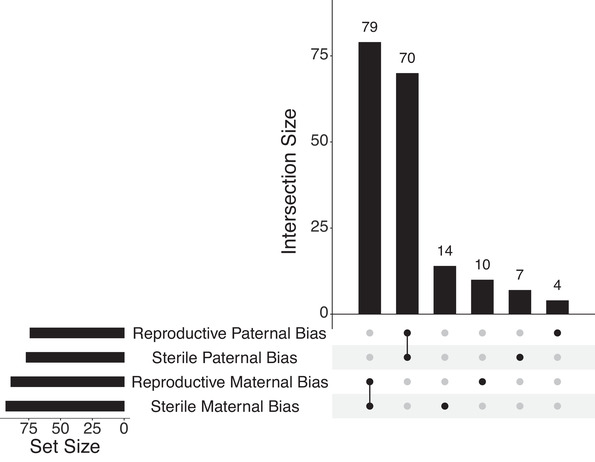
Overlapping genes showing parent‐of‐origin expression in reproductive and sterile workers. The set size indicates the number of genes in each list. The intersection size shows how many genes the corresponding lists have in common. A single dot refers to the number of genes unique to each list.

Reproductive and sterile workers share a significant number of genes showing parent‐of‐origin expression with the same parental bias (Fig. 4, maternal expression bias: hypergeometric test, $p=9.20\times \;10^{-108}$, paternal expression bias: hypergeometric test, $p=7.66\times \;10^{-90}$). The majority of genes identified as showing parental expression bias show the same bias in both abdomen and head tissue as well as across behaviorally defined phenotypes (dominant and subordinate reproductives and sterile foragers and nurses); see Supporting Information 2.0, Figs. S3– S8. There were no genes with maternal/paternal bias in one phenotype, which also had the opposite bias in the other phenotype. However, we did identify a small number of genes that show parent‐of‐origin expression in only one reproductive phenotype with the other phenotype showing bi‐parental expression. We found double the number of maternally expressed genes compared to paternally expressed genes unique to either reproductive or sterile workers (Fig. 4).

Overall genes showing parent‐of‐origin expression, including both maternal and paternal bias, have enriched GO terms for multiple biological processes (Supporting Information 1.1.2), specifically the GO terms “negative regulation of reproductive processes” (GO:2000242) and “female germ‐line sex determination” (GO:0019099) are enriched. Genes with either maternal or paternal bias in both reproductive and sterile workers also have enriched GO terms for multiple biological processes (Supporting Information 1.1.3 and 1.1.4). Specifically, paternally expressed genes in both reproductive and sterile workers are enriched for the GO term; “behavior” (GO:0007610).

GO terms for genes showing parent‐of‐origin expression in only reproductive or sterile workers were also enriched for various biological processes (Supporting Information 1.1.5). Including “histone ubiquitination” (GO:0016574) and “histone H2A monoubiquitination” (GO:0035518) in reproductive paternally expressed genes and “positive regulation of transcription” (GO:0045893) in maternally expressed genes.

### DIFFERENTIAL EXPRESSION BETWEEN REPRODUCTIVE AND STERILE WORKERS

To determine if genes that show parent‐of‐origin expression are involved in the generation of the reproductive phenotype differentially expressed genes between reproductive and sterile workers were identified. Differentially expressed genes were assessed separately for each tissue type as tissue explains the majority of variation within all of the RNA‐Seq samples (Supporting Information 2.0, Fig. S9).

Following differential expression analysis a total of 3505 genes were upregulated in the abdomen of reproductive workers compared to sterile workers and 4069 genes were downregulated (q<0.01) (Supporting Information 1.0.2). The enriched GO terms for the differentially expressed genes between reproductive and sterile workers in the abdomen included mostly regulatory processes but also “reproduction” (GO:0000003) (Supporting Information 1.0.3). Enriched GO terms associated specifically with upregulated genes in reproductive workers in the abdomen also included “reproduction” (GO:0000003) and “DNA methylation” (GO:0006306) (Supporting Information 1.0.4), these terms were not found in the enriched GO terms for genes upregulated in sterile workers (Supporting Information 1.0.5).

Considerably, fewer genes were differentially expressed in the head samples; 86 upregulated genes in reproductive compared to sterile workers and 41 downregulated genes (q<0.01) (Supporting Information 1.0.6). The majority of the GO terms associated with these differentially expressed genes involved biosynthetic processes (Supporting Information 1.0.7). Upregulated genes in the head tissue of reproductive workers also included “reproduction” (GO:0000003), whereas the upregulated genes in the head tissue of sterile workers consisted of mostly metabolic processes (Supporting Information 1.0.8 and 1.0.9).

### OVERLAP OF GENES WITH DIFFERENTIAL EXPRESSION AND PARENT‐OF‐ORIGIN EXPRESSION

We checked for overlap between genes showing parent‐of‐origin expression and genes that are differentially expressed between reproductive phenotypes. Genes showing maternal parent‐of‐origin expression in both phenotypes are enriched for genes which are also differentially expressed in head tissue (Supporting Information 2.0, Fig. S10; hypergeometric test, P = 0.004). Specifically *Serine Protease Inhibitor 3/4* (LOC100652301) shows maternal expression bias in both reproductive and sterile workers and is upregulated in the head tissue of reproductive workers. Genes with paternal parent‐of‐origin expression in both phenotypes do not significantly overlap with differentially expressed genes in head tissue (Supporting Information 2.0, Fig. S10; hypergeometric test, P = 0.148). There is also no significant overlap between genes showing parent‐of‐origin expression bias in both reproductive and sterile workers and differential expression in the abdomen (Supporting Information 2.0, Fig. S11; hypergeometric test, P = 0.996).

**Figure 5:**
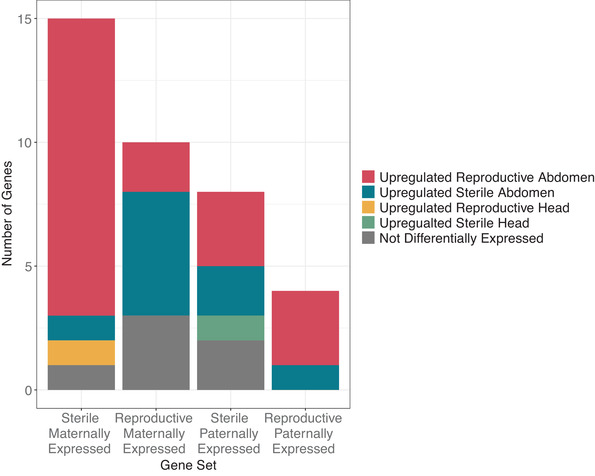
The number of genes which show parent‐of‐origin expression in one reproductive state and are also differentially expressed. Component bar chart showing the differential expression state of all genes that show parent‐of‐origin expression in either reproductive or sterile workers only. There is one gene that shows maternal expression bias in sterile workers, which is upregulated both in reproductive abdomen and head tissue. There is also one gene that shows paternal expression bias in sterile worker, which is upregulated in the head of sterile workers and the abdomen of reproductive workers. These two genes are therefore shown twice in the graph.

We did, however, find a large overlap between genes that show parent‐of‐origin expression in just one reproductive state and genes that are differentially expressed between reproductive states (Fig. 5), the majority of which are differentially expressed in the abdomen. Specifically, we find genes that are maternally expressed in one worker type generally show higher expression in the opposite phenotype where diploid expression is present. While the function of these genes and the related GO terms are diverse (Supporting Information 1.1.7), we do find genes involved in transcriptional regulation, which may indicate genes that display parent‐of‐origin expression are involved in regulating gene networks.

### HONEY BEE HOMOLOGY

To identify possible evolutionary conservation of imprinted genes in Hymenoptera, we compared the gene identified here to those previously identified in the honey bee (Galbraith et al. 2016). A custom database of putative orthologs was made between *A. mellifera* and *B. terrestris* in Marshall et al. (2019), containing 6539 genes. Sixty‐eight percent of differentially expressed abdominal genes were identified within the database, 43% of differentially expressed head genes, and 46% of genes showing parent‐of‐origin expression bias (Supporting Information 1.1.6). Gene lists were obtained from Galbraith et al. (2016), the ortholog database contained 38% of the differentially expressed genes identified between honey bee reproductive worker phenotypes and 53% of the genes found to show parent‐of‐origin expression bias (Supporting Information 1.1.6).

There was no significant overlap between the genes identified as showing parent‐of‐origin expression between both studies (hypergeometric P = 0.64), with only two genes overlapping (Supporting Information 2.0, Fig. S12). One of these is uncharacterized in both species (honey bee id: LOC552195, bumblebee id: LOC100648162) and the second is a serine protease inhibitor (honey bee id: LOC411889, bumblebee id: LOC100644680). The serine protease inhibitor shows paternal expression bias in honey bees and maternal bias in both bumblebee reproductive and sterile workers. It is not differentially expressed in the honey bee but it shows upregulation in the abdomen tissue of reproductive bumblebee workers compared to sterile workers. Additionally, Galbraith et al. (2016) identified numerous genes of interest, which are involved in reproductive behavior (*vitellogenin, yolkless, ecdysone‐receptor, and ecdysone‐induced protein*), which show paternal expression bias in honey bees, none of which show significant parent‐of‐origin expression in the bumblebee (Supporting Information 2.0, Fig. S13).

There was also no significant overlap between differentially expressed genes identified in head tissue and abdomen tissue between *B. terrestris* reproductive workers with those identified as differentially expressed between reproductive workers of *A. mellifera* from Galbraith et al. (2016) (Supporting Information 2.0, Fig. S14; head: hypergeometric *p* = 1, abdomen hypergeometric *p* = 1). This indicates that these species may use different mechanisms to initiate the reproductive phenotype in workers. If imprinted genes are involved in reproduction, this may explain the lack of overlap between genes showing parent‐of‐origin expression between these two species.

## Discussion

Using parental genome sequencing and offspring RNA‐seq, we have identified genes showing parent‐of‐origin allele‐specific expression in a primitively eusocial bumblebee species. There was no difference in the number of genes showing maternal or paternal expression bias in either reproductive or sterile workers. The genes showing the highest proportion of expression bias were all maternally expressed and there were double the number of genes showing maternal expression bias compared to paternal expression bias, which were unique to each worker phenotype. Additionally, reproductive‐related GO terms were enriched in both maternally and paternally biased genes.

Reproductive and sterile workers were chosen to provide a robust test for the kinship theory. It is possible that imprinted genes may maintain their expression bias regardless of the current reproductive state of the individual, that is, higher matrigenic expression compared to patrigenic expression may be present in queenless workers regardless of whether they have become reproductive or remained sterile. We have found the majority of genes showing that parent‐of‐origin expression are indeed conserved between reproductive and sterile workers. However, there are some which are unique to each reproductive state, indicating that these genes have become biallelically expressed during the transition from sterile to reproductive (in the case of genes showing parent‐of‐origin expression in sterile workers) or switched from biallelic expression to allele‐specific expression (in the case of genes showing parent‐of‐origin expression only in reproductive workers). These genes, which show that differential parent‐of‐origin specific expression between reproductive states, are therefore the candidate genes for influencing this phenotype. We do indeed find that the vast majority of these genes are differentially expressed between worker types indicating a possible regulatory role. While these genes are not involved directly in worker reproduction, they may influence gene expression networks that generate the later phenotype.

As in Galbraith et al. (2016), we also found no significant overlap of paternally expressed genes, common to both reproductive states, with differentially expressed genes between reproductive and sterile workers. However, we did find a significant overlap with maternally expressed genes present in both phenotypes and genes differentially expressed in head tissue between reproductive and sterile workers. This significant overlap should be interpreted cautiously, however as only 7 of the 103 unique maternally biased genes were differentially expressed in head tissue between reproductive phenotypes. The lack of overlap of differentially expressed genes with paternally expressed genes and the small overlap with maternally expressed genes suggests parent‐of‐origin expression may not *directly* influence reproductive status in bumblebee workers.

One of the overlapping genes found to be differentially expressed in head tissue between reproductive phenotypes, which also shows maternal expression bias is a serine protease inhibitor. One of the two homologous genes identified as showing parent‐of‐origin expression in this study and in honey bees was also a serine protease inhibitor. Serine proteases (also known as serpins) have been shown to be involved in insect immunity in various species including; the silkworm *Bombyx mori* (Zou et al. 2009), another species of silk producing moth *Antheraea pernyi* (Yu et al. 2017), and the mosquito *Anopheles gambiae* (Gorman and Paskewitz 2001). Most recently, a kazal‐type serine protease inhibitor has been directly linked to oocyte development in the desert locust *Schistocerca gregaria* (Guo et al. 2019). Future work identifying the function of serine protease inhibitors in social insects is needed to better understand the function of parent‐of‐origin expression of these genes in both *B. terrestris* and *A. mellifera*.

We found genes involved in histone modifications to be paternally expressed in reproductive workers. Histone modifications have been identified as an imprinting mark in plants (Rodrigues and Zilberman 2015) and thought to be involved in imprinting maintenance in mammals (Delaval and Feil 2004). Histone modifications can alter gene expression by affecting gene accessibility via chromatin (Bannister and Kouzarides 2011). Chromatin modifications have been associated with parent‐of‐origin expression in the fruit fly *Drosophilia melanogaster* (Joanis and Lloyd 2002). Galbraith et al. (2016) also found genes involved in histone modifications showing parent‐of‐origin expression in the honey bee.

Only two genes were found in common between those found in Galbraith et al. (2016) as showing parent‐of‐origin gene expression in the honey bee *A. mellifera* and those identified here in *B. terrestris*. Galbraith et al. (2016) used ovaries and fat bodies as their tissues samples, whereas we selected to test whole head and whole abdomen samples for strong signals of expression bias. Some imprinted genes in mammals are known to be tissue‐specific, such as *GRB10* which has been found to be maternally expressed in brain and muscle tissue but not in growth plate cartilage (McCann et al. 2001). Tissue specificity could account for the lack of concordance in parentally expressed genes found between *B. terrestris* and *A. mellifera*. Additionally, 51% of bumblebee genes and 52% of honey bee genes were not present in the homology database created.

Imprinted genes in mammals show much more consistency across species, with mice and humans reportedly sharing around 50 imprinted genes of around 150 and 100 characterized in each, respectively (Babak et al. 2015). Additionally, domestic cattle and pigs have been shown to share 14 imprinted genes of 26 and 18, respectively (Tian 2013). However, imprinted genes in plants generally show less conservation, with one study reporting 14% of maternally expressed genes and 29% of paternally expressed genes in *Capsella rubella* show the same imprinting status in *Arabidopsis thaliana*, even though both species belong to the Brassicaceae family (Hatorangan et al. 2016). Hatorangan et al. (2016) suggest that the lack of consistency between species could be the result of a historical shift in mating‐systems. Given the differences in mating systems between honey bees and bumblebees, and that variable predictions from the kinship theory apply to each species, rapid evolution of imprinted genes in Hymenoptera is a feasible explanation for the lack of consistency in potentially imprinted genes identified here and in honey bees.

The GO terms are associated with both maternally and paternally expressed genes are diverse. It has been suggested that imprinted genes can function as a mechanism for plasticity, allowing gene regulation to change depending on environmental conditions by activating the silenced allele and increasing dosage of that gene (Radford et al. 2011). Social insects display, sometimes extreme, phenotypic plasticity, where multiple discrete phenotypes (castes) can arise from a single genome within a colony. In some species this is genetically determined (Mott et al. 2015), however there is growing evidence epigenetic factors may play a role in caste determination in some species (Lyko et al. 2010; Bonasio et al. 2012; Marshall et al. 2019). Matsuura et al. (2018) modeled a genomic imprinting mediated caste determination system in the termite *R. speratus* and found this better explained the influence of parental phenotype on offspring than a purely genetic model. Given the diversity of genes found here showing both maternal and paternal expression bias we believe, along with Matsuura (2019), that further experimental investigation into the role of genomic imprinting in caste determination in social insects is needed.

The identification of genes showing parent‐of‐origin expression in this study lays the ground work for future research to identify potential epigenetic mechanisms of allele‐specific expression in social insects. Genes showing allele‐specific expression and DNA methylation have been previously identified in *B. terrestris* (Lonsdale et al. 2017), and genes involved in the reproductive process have been shown to be differentially methylated between reproductive phenotypes (Amarasinghe et al. 2014; Marshall et al. 2019). DNA methylation is the mechanism by which some genes are imprinted in mammals and plants (Scott and Spielman 2006) and so investigation of parent‐of‐origin methylation in *B. terrestris* may be fruitful.

These results provide support for Haig's kinship theory. As predicted, we have identified genes that show parent‐of‐origin expression, some of which are involved in reproductive processes. The most extreme bias was found in matrigenically biased genes. We also identified a small number of genes that show parent‐of‐origin expression in only sterile or reproductive workers and biparental expression in the other phenotype, the majority of which show maternal allele expression bias. Some of these genes are involved in transcriptional regulation and as such provide a candidate “master‐switch” for a cascade of gene expression changes, which result in the reproductive worker phenotype. This study therefore provides novel, independent support for this important evolutionary theory. The results of this study create a base for many future avenues of research including gene function analysis of serine protease inhibitors in Hymenoptera, epigenetic mechanisms of imprinting in insects and imprinted genes as a mechanism for plasticity, caste determination, and social evolution.

## AUTHOR CONTRIBUTIONS

T.W. and E.B.M. conceived the study. The reciprocal crosses were carried out by Biobest (Westerlo, Belgium) under supervision of F.W. J.S.Z. and K.B. carried out the behavioral observations and laboratory work. H.M. and T.W. carried out the analyses with input from A.V.G. H.M. and E.B.M. wrote the initial manuscript. All authors contributed to and reviewed the manuscript.

## DATA ARCHIVING

All RNA‐seq and whole genome sequencing data are available under NCBI BioProject number PRJNA329487. Custom scripts are available at the following doi:https://zenodo.org/record/3235636.

1

Associate Editor: S. Wright

## Supporting information


**Data S1**.

**Figure S1**: Boxplot showing the mean proportion of maternal expression for both reproductive and sterile workers for both tissue types and all colonies, for: all genes (n = 14,824), those which show no significant expression bias (n = 12,906), those with significant maternal (n = 723) or paternal expression bias (n = 1195) (q <0.05) and those with significant maternal (n = 160) or paternal (n = 122) expression bias as well as having a mean maternal expression bias >0.6 or <0.4.
**Figure S2**: Scatter plot showing the standard deviation for each gene plotted against the mean proportion of maternal expression.
**Figure S3**: The proportion of maternal expression for each tissue type of all genes found to show significant parental expression bias.
**Figure S4**: The proportion of paternal expression for each tissue type of all genes found to show significant parental expression bias.
**Figure S5**: The proportion of maternal expression for each behavioural reproductive type for all genes found to show significant parental expression bias.
**Figure S6**: The proportion of paternal expression for each behavioural reproductive type for all genes found to show significant parental expression bias.
**Figure S7**: The proportion of maternal expression for each behavioural reproductive type per tissue type for all genes found to show significant parental expression bias.
**Figure S8**: The proportion of paternal expression for each behavioural reproductive type per tissue type for all genes found to show significant parental expression bias.
**Figure S9**: PCA plot based on gene expression data. Samples separate by tissue type along PC1 and by reproductive status along PC2.
**Figure S10**: Overlapping genes showing parent‐of‐origin expression in reproductive (queen‐right) and sterile (queen‐less) workers with genes showing differential expression in head tissue.
**Figure S11**: Overlapping genes showing parent‐of‐origin expression in reproductive (queen‐right) and sterile (queen‐less) workers with genes showing differential expression in abdomen tissue.
**Figure S12**: Overlapping genes showing parent‐of‐origin expression in reproductive (queen‐right) and sterile (queen‐less) workers of *B*.
**Figure S13**: Maternal expression proportion by worker caste, sterile and reproductive.
**Figure S14**: Overlapping genes showing differential expression between reproductive (queen‐right) and sterile (queen‐less) workers of *B*.
